# The impact of the CERTAIN clinical decision support tool for structured intensive care unit admission and rounding is patient sex-independent: a secondary analysis of CERTAIN *

**DOI:** 10.62675/2965-2774.20250017

**Published:** 2025-09-09

**Authors:** Pien Swart, Aysun Tekin, Yue Dong, Marija Vukoja, Rahul Kashyap, Ognjen Gajic, Frederique Paulus, Marcus J. Schultz, for the CERTAIN - investigators of the Society of Critical Care Medicine Discovery Network

**Affiliations:** 1 Amsterdam University Medical Center - location AMC Department of Intensive Care Amsterdam Netherlands Department of Intensive Care, Amsterdam University Medical Center - location AMC - Amsterdam, Netherlands.; 2 Division of Pulmonary and Critical Care Medicine, Mayo Clinic Department of Medicine Rochester Minnesota United States Department of Medicine, Division of Pulmonary and Critical Care Medicine, Mayo Clinic - Rochester, Minnesota, United States.; 3 Mayo Clinic Department of Anesthesiology and Perioperative Medicine Rochester Minnesota United States Department of Anesthesiology and Perioperative Medicine, Mayo Clinic - Rochester, Minnesota, United States.; 4 University of Novi Sad Faculty of Medicine Novi Sad Vojvodina Serbia Faculty of Medicine, University of Novi Sad - Novi Sad, Vojvodina, Serbia.; 5 Mahidol University Mahidol-Oxford Tropical Research Unit Bangkok Thailand Mahidol-Oxford Tropical Research Unit, Mahidol University - Bangkok, Thailand.; 6 University of Oxford Nuffield Department of Medicine Oxford United Kingdom Nuffield Department of Medicine, University of Oxford - Oxford, United Kingdom.

**Keywords:** Critical care, Sex, Inequity, Quality improvement global health, Middle-income countries, Intensive care units

## Abstract

**Objective:**

Implementing the Checklist for Early Recognition and Treatment of Acute Illness and Injury (CERTAIN) decision support tool for structured intensive care unit admission and rounding was associated with an increased adherence to best care practices. We determined whether this association was patient sex-dependent.

**Design:**

Post hoc analysis of CERTAIN.

**Setting:**

prospective multinational quality improvement study.

**Patients:**

Adult patients admitted to one of the participating intensive care units.

**Interventions:**

Implementation of the CERTAIN decision support tool.

**Measurements:**

We compared incidence rates of omission of delivery of ten best care practices, including deep vein thrombosis and peptic ulcer prophylaxis, head of bed elevation, daily oral care, spontaneous breathing trials, family conferences, assessment of need for central lines and urinary catheters, and prescription of antimicrobials and sedation, between sexes, before and after implementation of the decision support tool. In addition, we determined whether sex differences existed amongst high-and middle-income countries.

**Main results:**

CERTAIN comprised a total of 4,256 patients, with 588 females and 859 males before the implementation of the decision support tool and 1,169 females and 1,640 males after its implementation. Overall, there was no notable difference in care between sexes, neither before nor after implementation, and both sexes in high-income and middle-income countries experienced equal benefits from checklist implementation.

**Conclusion:**

The impact of a clinical decision support tool for structured intensive care unit admission and rounding on adherence to best care practices showed minimal variation between sexes.

## INTRODUCTION

Numerous studies have highlighted the presence of sex inequity in critical care provision, with significant differences observed in the utilization of various interventions. Specifically, females tend to receive interventions such as deep venous thrombosis prophylaxis,^( 1 )^ vasoactive medication,^( 2 - 4 )^ fluid administration, transfusion and renal replacement therapy,^( 1 - 3 , 5 - 7 )^ respiratory support,^( 1 - 5 , 8 - 10 )^ neuromonitoring,^( 2 )^ catheter use^( 1 , 2 , 5 , 8 )^ and antibiotic therapy^( 7 , 11 )^ less frequently than males. There is also a higher incidence of care limitations among females.^( 1 , 12 , 13 )^

A conveniently-sized multicontinental study, named Checklist for Early Recognition and Treatment of Acute Illness and Injury (CERTAIN), showed that implementation of a clinical decision support tool for structured intensive care unit (ICU) admission and rounding was associated with an increased adherence to important best care processes.^( 14 )^ In a subgroup analysis, these improvements were notably more substantial in middle-income countries (MIC) than in high-income countries (HIC).

It is uncertain whether the implementation of CERTAIN had comparable effects on females and males. Therefore, we reanalyzed the CERTAIN database to test the hypothesis that the effects of implementation would be different between sexes. In a subgroup analysis, we also assessed the impact of implementation on females and males in HIC and MIC.

## METHODS

### Institutional Review Board statement

The Institutional Review Board of the Mayo Clinic, Rochester, Minnesota, United States (12-007998) approved the study protocol, and thereafter in all participating centers. Procedures were followed according to the ethical standards of the responsible committee on human experimentation (institutional or regional) and with the Helsinki Declaration of 1964 and its subsequent amendments or equivalent ethical standards.

### Design, setting, and participants

Post hoc analysis of CERTAIN, a pragmatic, prospective, exploratory, multicontinental, international, multicenter, before-after quality-improvement study in 34 ICUs in 5 HIC and 10 MIC, conducted between November 2013 and December 2017.^( 14 )^ The study was registered at clinicaltrials.gov (identifier NCT01973829). Informed consent was obtained from all individual participants included in the study.

Patients were eligible if admitted to one of the participating ICUs and ≥ 18 years of age. Patients admitted only for monitoring, patients with a planned admission for routine postoperative surveillance for less than 24 hours after uncomplicated surgery, readmitted patients, and patients transferred from an ICU outside a study hospital were excluded. Patients without a recorded admission date or an incorrectly recorded date did not participate. No additional exclusion criteria were used for this post hoc analysis.

### Checklist

CERTAIN used a web-based or paper decision support tool displaying relevant clinical information, including the evidence-based checklist.^( 14 )^ The core of the intervention in CERTAIN was a structured approach to admission and daily rounding by using the checklist to prompt clinicians to follow best care practices.

### Data collected

Data was collected for 3 months before and 6 months after implementing the decision support tool and included ICU characteristics, patient demographics, baseline characteristics, comorbidities, and limitations on life support interventions. The Sequential Organ Failure Assessment (SOFA) score was collected for each patient. Daily care processes were recorded on the day of ICU admission (calendar day 0) and on calendar days 1, 2, 3, 7, 14, and 21 for as long as the patient remained in the ICU and included ten best care practices: Did the patient receive (1) deep vein thrombosis and (2) peptic ulcer prophylaxis?; Was there (3) head of bed elevation?; Is the (4) daily oral care performed?; Is there a documented assessment of (5) a spontaneous breathing trial?; Is there a documented (6) family conference or discussion?; Was there an assessment of the need for (7) central lines and (8) urinary catheters?; Was there a documented assessment to continue or discontinue the current prescription of (9) antimicrobials and current (10) sedation medication? Adherence to these daily care processes was recorded for each observation day as either "Yes" or "No". Patients were followed to hospital discharge to assess death in the ICU or hospital and duration of stay in ICU and hospital.

### Definitions

Non-adherence to daily care processes was quantified as an incidence rate, defined as the ratio of the number of observations of not receiving basic care procedures among eligible patients (events) to the number of total observations in which the specific intervention was indicated (exposure) expressed per 1,000 days of specific intervention. Since we recorded biological sex rather than gender identity, we use the term "sex" to align with the available documentation and data structure.

### Endpoints

We used the same endpoints as in the primary analysis of CERTAIN, i.e., the omission of delivery of the abovementioned best care practices; ICU, hospital, and mortality, and ICU and hospital length of stay served as secondary endpoints.

### Sample-size calculation

No sample-size calculation was performed for this current analysis. The sample size was based on the number of patients available in the database.

### Statistical analysis plan

Descriptive statistics were used to report differences between groups. Continuous distributed variables are expressed as medians and their interquartile ranges, and categorical variables are expressed as frequencies and proportions. Fisher's exact tests were used for categorical variables, and the Wilcoxon rank-sum test was used for continuous variables.

The cohort was divided into two groups: females and males. For all analyses, males were used as the reference group.

Nonadherence (the "No" outcome) was chosen as the endpoint. For nonadherence to the ten best care practices, we ignored day 0, which was the day of ICU admission before and after implementation of the decision support tool. Sexes were compared concerning the primary endpoint using Poisson regression. Then, to adjust for a center effect, the incidence rate ratio was calculated with the center modeled as a random effect in a generalized linear mixed model.

Logistic regression was used to compare mortality rates, and linear regression using geometric means was used to evaluate hospital and ICU length of stay, both before and after the checklist's implementation. A generalized linear mixed-effects model with the center modeled as a random effect and mechanical ventilation, life support limitation, and comorbidity as fixed effects was used.

The analyses were repeated in the subgroups from HIC and MIC. Country income status was defined by the World Bank 4. High-income countries included Croatia, Ireland, Poland, Saudi Arabia, and the United States. Middle-income countries included Bosnia and Herzegovina, China, India, Lebanon, Mexico, Pakistan, Philippines, Serbia, Tanzania, and Turkey.

All analyses were conducted in R version 4.0.3 (R Foundation for Statistical Computing (https://www.R-project.org/), Vienna, Austria); statistical significance was set at p value < 0.05. Details are provided in the Supplementary Material.

## RESULTS

### Patients

**Figure 1:**
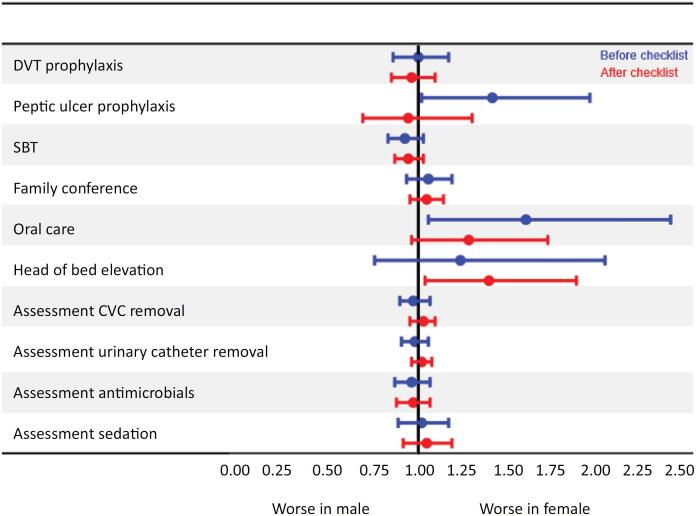
The incidence rates of omissions in daily care processes between the sexes - middle-income countries + high-income countries. Checklist for Early Recognition and Treatment of Acute Illness and Injury (CERTAIN). An event refers to a non-adherence to an evidence-based Daily Care Process. Adjusted for center effect incident rate ratios. In this figure, the blue whisker compares the incident rates of events between female *versus* male patients before the checklist, and the red line compares the incident rates of events after the checklist.

The analysis included 4,256 patients, 588 and 1,169 females and 859 and 1,640 males before and after implementing the decision support tool, respectively ( Figure 1S - Supplementary Material ). Females were older and had a lower median body weight but were more often obese ( Table 1 ). Females more often had a history of hypothyroidism, rheumatoid arthritis, and collagen vascular disease; males more often had a history of alcohol abuse and liver disease. Compared to patients in HIC, patients in MIC were younger and shorter and had a lower median SOFA score ( Tables 1SA and 1SB - Supplementary Material ). Patients in HIC more often had a history of alcohol abuse.

**Table 1:** Patient demographics and baseline characteristics

		Before			After		
		Female n = 588	Male n = 859	p value	Female n = 1,169	Male n = 1,640	p value
Age		64.1 [48.5 - 77.5]	60.2 [44.3 - 73.0]	< 0.001	62.7 [46.3 - 75.8]	61.3 [46.2 - 73.5]	0.167
Weight		63.0 [54.0 - 75.0]	72.0 [65.0 - 86.6]	< 0.001	64.0 [55.0 - 75.0]	73.0 [65.0 - 84.0]	< 0.001
Height		160.0 [155.0 - 165.0]	172.0 [168.0 - 176.5]	< 0.001	160.0 [155.0 - 165.0]	170.0 [167.0 - 176.0]	< 0.001
Admission from				0.611			0.476
	Home	283 (48.1)	397 (46.3)		684 (58.6)	921 (56.2)	
	Nursing home	14 (2.4)	21 (2.4)		16 (1.4)	23 (1.4)	
	ED	186 (31.6)	258 (30.1)		328 (28.1)	463 (28.2)	
	Outside hospital ED	82 (13.9)	146 (17.0)		103 (8.8)	177 (10.8)	
	Other	23 (3.9)	36 (4.2)		36 (3.1)	55 (3.4)	
Life support limitation		83 (14.1)	134 (15.6)	0.455	145 (12.6)	140 (8.7)	0.001
SOFA		6.0 [3.0 - 9.0]	7.0 [4.0 - 10.0]	< 0.001	6.0 [3.0 - 9.0]	6.0 [4.0 - 9.0]	0.016
Mechanical ventilation		320 (54.7)	502 (58.6)	0.144	673 (58.1)	1007 (62.0)	0.037
Comorbidities							
	Congestive heart failure	112 (19.0)	158 (18.4)	0.784	162 (13.9)	214 (13.0)	0.537
	Cardiac arrhythmias	95 (16.2)	106 (12.3)	0.044	126 (10.8)	170 (10.4)	0.755
	Valvular disease	41 (7.0)	52 (6.1)	0.513	54 (4.6)	56 (3.4)	0.114
	Pulmonary circulation disorders	56 (9.5)	79 (9.2)	0.854	84 (7.2)	109 (6.6)	0.597
	Hypertension	274 (46.6)	369 (43.0)	0.178	522 (44.7)	682 (41.6)	0.113
	Paralysis	14 (2.4)	45 (5.2)	0.007	34 (2.9)	34 (2.1)	0.171
	Other neurologic disorders	54 (9.2)	102 (11.9)	0.120	82 (7.0)	124 (7.6)	0.608
	Diabetes, uncomplicated	100 (17.0)	139 (16.2)	0.719	181 (15.5)	214 (13.0)	0.069
	Diabetes, complicated	67 (11.4)	78 (9.1)	0.155	139 (11.9)	179 (10.9)	0.433
	Hypothyroidism	31 (5.3)	17 (2.0)	0.001	71 (6.1)	40 (2.4)	< 0.001
	Renal failure	88 (15.0)	141 (16.4)	0.465	153 (13.1)	206 (12.6)	0.688
	Liver disease	30 (5.1)	81 (9.4)	0.002	56 (4.8)	86 (5.2)	0.602
	Peptic ulcer disease excluding bleeding	15 (2.6)	25 (2.9)	0.746	21 (1.8)	27 (1.6)	0.770
	AIDS	4 (0.7)	5 (0.6)	1.000	1 (0.1)	11 (0.7)	0.019
	Lymphoma	8 (1.4)	7 (0.8)	0.429	19 (1.6)	30 (1.8)	0.771
	Metastatic cancer	31 (5.3)	33 (3.8)	0.196	53 (4.5)	87 (5.3)	0.380
	Solid tumor without metastasis	30 (5.1)	50 (5.8)	0.640	91 (7.8)	105 (6.4)	0.176
	Rheumatoid arthritis/collagen vascular disease	19 (3.2)	10 (1.2)	0.007	39 (3.3)	16 (1.0)	< 0.001
	Coagulopathy	23 (3.9)	31 (3.6)	0.779	34 (2.9)	47 (2.9)	1.000
	Obesity	64 (10.9)	60 (7.0)	0.010	72 (6.2)	51 (3.1)	< 0.001
	Weight loss	26 (4.4)	36 (4.2)	0.895	36 (3.1)	43 (2.6)	0.489
	Fluid and electrolyte disorders	63 (10.7)	106 (12.3)	0.360	98 (8.4)	140 (8.5)	0.945
	Blood loss anemia	15 (2.6)	35 (4.1)	0.143	45 (3.8)	57 (3.5)	0.610
	Deficiency anemia	31 (5.3)	32 (3.7)	0.189	36 (3.1)	25 (1.5)	0.008
	Alcohol abuse	8 (1.4)	57 (6.6)	< 0.001	17 (1.5)	73 (4.5)	< 0.001
	Drug abuse	7 (1.2)	16 (1.9)	0.394	13 (1.1)	33 (2.0)	0.071
	Psychosis	9 (1.5)	15 (1.7)	0.836	20 (1.7)	20 (1.2)	0.333
	Depression	27 (4.6)	32 (3.7)	0.420	41 (3.5)	34 (2.1)	0.024
	Other	158 (26.9)	232 (27.0)	1.000	319 (27.3)	384 (23.4)	0.022
	None	34 (5.8)	54 (6.3)	0.738	135 (11.5)	227 (13.8)	0.077

ED - emergency department; SOFA - Sequential Organ Failure Assessment. Data are medians (interquartile range) or n (%). Percentages may not total 100 because of rounding.

### Nonadherence to best care practices

Prior to implementation, nonadherence to best care practices did not vary significantly between sexes, except for peptic ulcer prophylaxis and daily oral care, which were more often omitted in females ( Figure 1 and Table 2SA [ Supplementary Material ]). After implementation, head of bed elevation was more often omitted in female patients ( Figure 1 and Table 2SB [Supplementary Material] ).

DVT - deep vein thrombosis; SBT - spontaneous breathing trials; CVC - central venous catheter.

**Figure 2:**
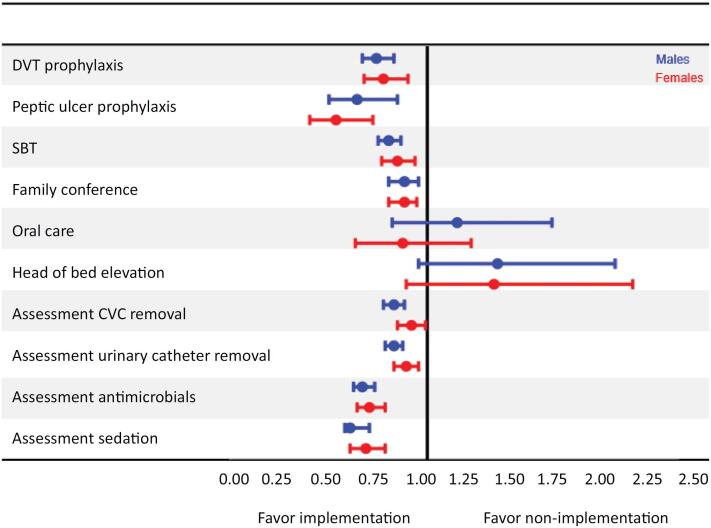
The incidence rates of omissions in daily care processes before *versus* after the implementation. Checklist for Early Recognition and Treatment of Acute Illness and Injury (CERTAIN). Adjusted for center effect incident rate ratios. In this figure the blue whisker compares the incident rates of events between before versus after the checklist in male patients and the red line compares the incident rates of events in female patients.

The effects of implementing the checklist were not different between sexes ( Figure 2 ). After implementation, there were no differences observed in ICU, hospital, mortality, and hospital length of stay between sexes ( Tables 2 and 3 ). However, the ICU length of stay was shorter in female patients ( Table 3 ).

DVT - deep vein thrombosis; **SBT - spontaneous breathing trials; CVC - central venous catheter.**

**Table 2:** Clinical outcomes: mortality

Before Total = 1,447	Male, n = 859 Mortality (%)	Death	Patient, n	Female, n = 588 Mortality (%)	Death	Patient, n	Effect measure (95%CI) Adjusted OR	p value
ICU mortality	28.7 (25.7 - 31.9)	240	836	29.0 (25.4 - 32.8)	166	573	1.04 (0.814 - 1.33)	0.76
Hospital mortality	34.3 (31.1 - 37.6)	284	829	34.0 (30.3 - 38.0)	193	567	0.996 (0.787 - 1.26)	0.98
28 days mortality	36.7 (33.5 - 40.1)	298	812	37.1 (33.2 - 41.2)	206	555	1.08 (0.856 - 1.37)	0.51
After Total = 2,809	Male, n = 1,640 Mortality (%)	Death	Patient, n	Female, n = 1,169 Mortality (%)	Death	Patient, n	Effect measure (95%CI) Adjusted OR	p value
								p
ICU mortality	23.9 (21.9 - 26.1)	386	1,613	22.8 (20.5 - 25.3)	262	1,149	0.916 (0.756 - 1.11)	0.37
Hospital mortality	29.2 (27.0 - 31.5)	470	1,609	28.1 (25.6 - 30.8)	323	1,148	0.917 (0.765 - 1.10)	0.35
28 days mortality	31.6 (29.4 - 33.9)	504	1,595	30.0 (27.4 - 32.7)	341	1,136	0.894 (0.748 - 1.07)	0.22

95%CI - 95% confidence interval; OR - odds ratio. ICU - intensive care unit.

**Table 3:** Clinical outcomes: intensive care unit and hospital length of stay

Before Total = 1447	Male, n = 859 Geometric mean (SD), d	Female, n = 588 Geometric mean (SD), d	Effect measure (95%CI) Adjusted ratio of geometric means	p value
ICU length of stay	6.63 (2.97)	6.34 (2.97)	0.98 (0.879 - 1.09)	0.72
Hospital length of stay	15.2 (3.1)	14.4 (2.99)	0.96 (0.857 - 1.07)	0.48
After Total = 2,809	Male, n = 1640 Geometric mean (SD), d	Female, n = 1169 Geometric mean (SD), d	Effect measure (95%CI) Adjusted ratio of geometric means	p value
ICU length of stay	6.12 (2.87)	5.34 (2.75)	0.909 (0.843 - 0.98)	0.013
Hospital length of stay	15 (2.81)	14 (2.84)	0.991 (0.919 - 1.07)	0.81

SD - standard deviation; 95%CI - 95% confidence interval; ICU - intensive care unit.

### High-income versus middle-income countries

The implementation of the checklist had consistent effects across the geo-economic settings ( Figures 2S to 5S and Tables 3S to 11S - Supplementary Material ).

## DISCUSSION

The findings of this post hoc analysis of CERTAIN can best be summarized as follows: before implementation of a clinical decision support tool for structured ICU admission and rounding, nonadherence to ten best care practices showed minimal variation between sexes; and females and males experienced equal benefits from checklist implementation. In addition, the checklist implementation yielded consistent outcomes across various geo-economic settings.

Our analysis has strengths. CERTAIN was a worldwide study with patients originating from ten MIC and five HICs across five continents, university-affiliated hospitals, and teaching and non-teaching centers, increasing the generalizability of our findings. The original study was large and robust and used broadly accepted evidence-based checklists, algorithms, and educational modules. The dataset was rich, with a negligible amount of missing data. The large number of patients allowed us to perform several analyses of MIC and HIC, including a subgroup analysis. An analysis plan, in place before performing the analysis, was strictly followed.

Our analysis did not reveal significant differences in care between the sexes except for peptic ulcer prophylaxis and daily oral care, which were more frequently omitted in females before implementing clinical decision support tools for structured ICU admission and rounding. This finding is in contrast to the findings of previous investigations.^( 1 - 13 )^ These prior studies showed important differences in care between the sexes. There is limited evidence suggesting a lower risk of peptic ulcers in women, particularly at a younger age,^( 15 )^ which may explain the more frequent omission of peptic ulcer prophylaxis in female patients. This difference, though, disappeared after the implementation of the checklist. Studies looking into sex differences in the omission of daily best care practices that were part of the tool used in this study are lacking. One study showed no significant association between the omission of early (< 24 hours) thromboprophylaxis with the patient's sex,^( 16 )^ and another study showed no sex difference in time for the head of the bed elevation.^( 17 )^ It is unclear why we did not find differences in best care practices between sexes. One possibility is that we are dealing with observer bias: even before the implementation of the checklist, i.e., in the first period of the study, there may have already been awareness to apply care as effectively as possible. However, we cannot rule out the possibility that overall care has improved, for example, through implementation processes prior to our study.

After implementing the clinical decision support tool for structured ICU admission and rounding, differences in peptic ulcer prophylaxis and daily oral care between the sexes had disappeared, with the head of the bed elevation emerging as a differentiating factor. However, it should be noted that the confidence intervals for these three practices were relatively broad, indicating a degree of imprecision in the data. Additionally, the observed difference in head of bed elevation was minimal, likely not significantly affecting outcomes.

After implementation of the checklist, female patients had a statistically significant shorter ICU length of stay compared to male patients. Several factors may explain this observation. One possibility is that the same quality improvement would benefit female patients more. Another possibility is that sex-specific differences in care delivery influenced ICU length of stay. The fact that this difference emerged only after checklist implementation suggests that the standardized decision-support tool may have improved basic ICU care, potentially benefiting female patients more. This could be due to bias mitigation, as the tool may have reduced unconscious biases in clinical decision-making, leading to more consistent and appropriate care for female patients. However, it remains possible that the statistically significant shorter ICU length of stay occurred by chance, and further research is needed to confirm whether this difference is reproducible.

In the HIC and MIC subgroup analysis, we found no sex differences in best-care practices and outcome measures. However, only in MIC did the implementation of the checklist lead to a decrease in outcome measures for both sexes, including mortality and ICU- and hospital length of stay. This suggests that while implementing the tool may have less potential to improve outcomes in HIC, it holds strong potential to benefit patients in countries with room for improvement in care. Possible reasons why the checklist did not result in decreased outcome measures in HIC could be attributed to the fact that these countries have more resources for quality improvement interventions and an established organizational safety culture. It is also plausible that baseline adherence to processes of care was higher in HIC or that fewer centers and patients were enrolled from these countries. Nonetheless, the results indicate that a systematic checklist is feasible in resource-limited settings.

Future research should explore sex differences in treatment. We need to expand research in precision medicine for both sexes in the ICU since addressing these differences is challenging given the heterogeneity of patients, including hormonal status and physiology, socially constructed preferences, care limitations, and diagnoses.

Our analysis has limitations. CERTAIN was a before-after observational study, which somewhat limits the ability to establish causality. Awareness of the study may have affected the impact of the intervention. The selection of ICUs relied on personal contacts, potentially introducing selection bias. Only hospitals willing to participate and motivated to enhance the quality of care and those with the necessary time and resources completed the study and were included in the analysis.^( 14 )^ Our analysis is susceptible to documentation bias, as adherence was determined from recorded compliance, which may not fully capture actual clinical practice. Best practices may have been followed but not consistently documented, potentially leading to underestimating adherence rates. Our data were collected between 2013 and 2017, which some may consider outdated. Clinical practices and protocols may have evolved. However, the fundamental principles of ICU care remain unchanged, and the key aspects of the studied interventions remain relevant today. Moreover, our primary research question focused on whether the checklist had comparable effects in female and male patients. Consistent with findings from other studies in various ICU cohorts, fewer female patients were admitted to the participating ICUs. Given our sample size, we do not anticipate this affecting the overall statistical power or the generalizability. While we adjusted for ventilation, life support limitations, and comorbidities, residual confounding may still be present. Not all relevant daily practices were collected and thus studied, including important bundles for catheter insertions, nutrition, or decubitus prevention. Furthermore, it is important to note that we had no data on pregnancy status. Staffing levels and logistical constraints were also not assessed daily, which may have influenced clinical practice. These factors should be considered when interpreting the findings. In our analysis, we have accounted for a center effect, which should have mitigated the effects of variations in resource availability and institutional practices. The findings may not be applicable to patients transferred from ICUs outside the participating hospital, as they were excluded from this study.^( 14 )^

Unfortunately, this study did not adequately capture cultural, traditional, and contextual factors. These elements may significantly influence healthcare practices, vary across geographic regions, and contribute to differences in care between sexes. More research is needed to understand their impact and ensure more comprehensive and equitable healthcare strategies.

## CONCLUSION

The impact of a clinical decision support tool for structured intensive care unit admission and rounding on adherence to ten best care practices showed only minor variations between females and males, and this observation remained consistent across intensive care units in various geo-economic areas.

## List of collaborators

**Table:** 

Names In Native Languages	Orcid	Institution	City	Country
Aijun Pan	https://orcid.org/0000-0002-3853-3879	AnHui Province Hospital	Hefei	China
Aida Mujakovic	https://orcid.org/0000-0002-0022-1482	General Hospital "Prim. dr Abdulah Nakas"	Sarajevo	Bosnia and Herzegovina
Alan Sustic	https://orcid.org/0000-0001-8393-4332	Clinical Hospital Center Rijeka	Rijeka	Croatia
Anna Kluzik	https://orcid.org/0000-0003-3865-300X	Heliodor Swiecicki Clinical Hospital at the Karol Marcinkowski Medical University	Poznan	Poland
Atilla Ramazanoglu	https://orcid.org/0000-0002-7215-6237	Akdeniz University Hospital	Antalya	Turkey
Aysen Erdogan		Süleyman Demirel University	Isparta	Turkey
Carolina L. Tapia		St. Luke's Medical Center	Metro Manila	Philippines
Chandan Dey		Ispat General Hospital	Rourkela	India
Cornelius Sendagire	https://orcid.org/0000-0002-9039-7325	Uganda Heart Institute	Kampala	Uganda
Dragana Markotic	https://orcid.org/0000-0002-6765-766X	University Clinical Hospital Mostar	Mostar	Bosnia and Herzegovina
Emily Naylor		St. James's Hospital	Dublin	Ireland
Erric Cinco		The Medical City Hospital	Metro Manila	Philippines
Fatima Ajaz		Shaukat Khanum Memorial Cancer Hospital and Research Center	Sindh	Pakistan
Feihu Zhou		Chinese PLA General Hospital	Beijing	China
Ganbold Lundeg	https://orcid.org/0000-0001-8470-7342	Central State Universtiy Hospital	Milledgeville	Mongolia
Haitao Lan	https://orcid.org/0000-0002-7198-6503	Guang'anmen Hospital	Beijing	China
Harpreet Singh		Lok Nayak Hospital, Maulana Azad Medical College	New Delhi	India
Ignacio Martin-Loeches	https://orcid.org/0000-0002-5834-4063	St. James's Hospital	Dublin	Ireland
Jianjun Gui		Dongguan Kanghua Hospital	Dongguan	China
Jose Guillermo Dominguez Cherit	https://orcid.org/0000-0003-1403-4415	Instituto Nacional de Ciencias Médicas y Nutrición Salvador Zubirán	Mexico City	Mexico
Jose Yunen	https://orcid.org/0009-0007-5479-6955	CEDIMAT, Plaza de la Salud	Santo Domingo	Dominican Republic
Jovan Matijasevic	https://orcid.org/0000-0002-2476-0763	The Institute for Pulmonary Diseases of Vojvodina	Novi Sad	Serbia
Juvelikian Georges		St. George's Hospital	Beirut	Lebanon
KB Chetak		JSS Hosptial	Mysore	India
Kenana Zejnilovic		University Clinical Center Sarajevo	Sarajevo	Bosnia and Herzegovina
Lejla Pasic	https://orcid.org/0000-0002-1447-0252	Cantonal Hospital "Dr. Safet Mujic"	Mostar	Bosnia and Herzegovina
Leonardo Rocha		Hospital Israelita Albert Einstein	Jardim Leonor	Brazil
Lin Dou	https://orcid.org/0009-0003-8604-1777	Tianjin First Central Hospital	Tianjin	China
Maja Surbatovic	https://orcid.org/0000-0002-7722-1245	Military Medical Academy	Belgrade	Serbia
Mandyam Dhali Ravi	https://orcid.org/0000-0002-6739-2278	JSS Hospital	Mysore	India
Manuel Hache-Marliere	https://orcid.org/0000-0002-2972-3208	Rush University	Chicago	United States
Mihailo Stojic		Military Medical Academy	Belgrade	Serbia
Min Shao	https://orcid.org/0000-0002-3748-1333	AnHui Province Hospital	Hefei	China
Mradul Daga	https://orcid.org/0000-0001-7774-7602	Lok Nayak Hospital, Maulana Azad Medical College	New Delhi	India
Oguz Kilickaya	https://orcid.org/0000-0003-0462-549X	Mayo Clinic Rochester	Rochester	United States
Otgontuya Gombo		Central State Universtiy Hospital	Milledgeville	Mongolia
Ozlem Cakin		Akdeniz University Hospital	Antalya	Turkey
Parvez Mir		Wyckoff Heights Medical Center	New York	United States
Paul Ufoegbunam		University of Nigeria Teaching Hospital	Enugu	Nigeria
Pedja Kovacevic	https://orcid.org/0000-0001-6654-5351	University Clinical Center of Republika Srpska	Banja Luka	Bosnia and Herzegovina
Rajyabardhan Pattnaik		Ispat General Hospital	Rourkela	India
Reina Suzuki		Jichi Medical University Saitama Medical Center	Saitama	Japan
Salim Surani		Corpus Christi Medical Center	Texas	United States
Sasa Dragic		University Clinical Center of Republika Srpska	Banja Luka	Bosnia and Herzegovina
Shouhong Wang		Guangdong Provincial People's Hospital	Guangzhou	China
Sixtus Ruyumbu		Mbeya Zonal Referral Hospital	Mbeya	Tanzania
Su Jung Choi		Instituto Nacional de Ciencias Médicas y Nutrición Salvador Zubirán	Mexico City	Mexico
Vakil Abhay		Corpus Christi Medical Center	Texas	United States
Varma Muralidhar		Kasturba Medical College Hospital	Karnataka	India
Wei Liu		Xiangya Hospital Central South University	Changsha	China
Xin Zhao		Guang'anmen Hospital	Beijing	China
Yan Kang		West China Hospital of Sichuan University	Chengdu	China
Yaseen Arabi		King Abdulaziz Medical City	Riyadh	Saudi Arabia
Yimin Li		The First Affiliated Hospital of Guangzhou Medical University	Guangzhou	China
ZhiGang Chang		Beijing Hospital	Beijing	China
