# Supplementary material for: The impact of the CERTAIN clinical decision support tool for structured intensive care unit admission and rounding is patient sex-independent: a secondary analysis of CERTAIN
Source: Crit Care Sci. 2025 Sep 9;37:e20250017. doi: 10.62675/2965-2774.20250017 (PMC12614940; doi:10.62675/2965-2774.20250017)
Supplement: Supplementary file 1 [file 2965-2774-ccsci-37-e20250017-Mat-suppl.pdf]

Critical Care Science
https://doi.org/10.62675/2965-2774.20250017

SUPPLEMENTARY MATERIAL

The impact of the CERTAIN clinical decision support tool for structured intensive care unit admission and rounding is patient sex-independent: a secondary analysis of CERTAIN*

Pien Swart[1], Aysun Tekin[2], Yue Dong[2,3], Marija Vukoja[4], Rahul Kashyap[2], Ognjen Gajic[2], Frederique Paulus[1], Marcus J. Schultz[1,5,6] for the CERTAIN* - investigators of the Society of Critical Care Medicine Discovery Network

*Checklist for Early Recognition and Treatment of Acute Illness and Injury

METHODS

Details statistical analysis

The Poisson distribution can be applied to data containing large numbers of rare possible events. In this study, nonadherence was the rare event.

The incidence rate ratio was calculated with the center modeled as a random effect in a generalized linear mixed model. The likelihood ratio test was used to determine the statistical significance of the incident rate ratio, using two models; a full model with center and sex; and a nested model with only center. The difference in log likelihood between the two models indicated the significance of the incident rate ratio (sex effect) adjusted for the centers.

Specifically, for mortality the dependent variable was 0/1 coded mortality and the independent variables were sex and the aforementioned patient characteristics. The likelihood ratio test was used to determine the statistical significance of the adjusted odds ratio, using the nested and full models. The analysis was conducted separately for intensive care unit mortality, hospital mortality, and 28-day mortality.

Specifically, for the length of stay the log transformed length of stay was the dependent variable; the independent variables were sex and the aforementioned characteristics. The model's sex coefficient is the log of the ratio of the geometric means. The values reported in the tables are the anti-log of the coefficient, which is a ratio of the geometric means. The F test comparing a nested and full model was used to determine the statistical significance of the adjusted ratio of geometric means.

This is an open access article under the CC BY license (https://creativecommons.org/licenses/by/4.0/).

Table 1SA - Before implementation: baseline characteristics low- and middle-income and high–income countries

	LMIC				HIC			
	Overall, n = 1,065	Female, n = 425 (40%)	Male, n = 640 (60%)	p value	Overall, n = 382	Female, n = 163 (43%)	Male, n = 219 (57%)	p value
Age	60.0 [42.8 - 73.7]	62.3 [44.6 - 75.8]	57.6 [42.3 - 72.2]	0.018	67.7 [55.1 - 77.6]	69.9 [57.8 - 79.2]	65.0 [52.1 - 74.6]	0.008
Weight	68.0 [59.0 - 80.0]	60.0 [51.0 - 70.0]	70.0 [63.4 - 81.5]	< 0.001	78.0 [65.0 - 95.0]	70.0 [58.3 - 88.4]	80.0 [70.0 - 96.8]	< 0.001
Height	168.0 [160.0 - 173.0]	160.0 [155.0 - 165.0]	172.0 [168.0 - 175.0]	< 0.001	168.0 [160.0 - 175.1]	160.0 [155.0 - 165.0]	175.0 [170.0 - 180.0]	< 0.001
Hospital admission source				0.107				0.544
Home	550 (51.6)	233 (54.8)	317 (49.5)		130 (34.1)	50 (30.7)	80 (36.7)	
Nursing home	22 (2.1)	8 (1.9)	14 (2.2)		13 (3.4)	6 (3.7)	7 (3.2)	
ED	287 (26.9)	117 (27.5)	170 (26.6)		157 (41.2)	69 (42.3)	88 (40.4)	
Outside hospital ED	169 (15.9)	52 (12.2)	117 (18.3)		59 (15.5)	30 (18.4)	29 (13.3)	
Other	37 (3.5)	15 (3.5)	22 (3.4)		22 (5.8)	8 (4.9)	14 (6.4)	
Life support limitations	163 (15.3)	62 (14.6)	101 (15.8)	0.664	54 (14.2)	21 (13.0)	33 (15.1)	0.656
SOFA	6.0 [4.0 - 9.0]	5.0 [3.0 - 8.0]	6.0 [4.0 - 10.0]	< 0.001	8.0 [4.0 - 11.0]	8.0 [4.0 - 10.0]	8.0 [5.0 - 11.0]	0.190
Mechanical ventilation	604 (56.9)	232 (54.8)	372 (58.2)	0.283	218 (57.5)	88 (54.3)	130 (59.9)	0.295
Comorbidities								
Congestive heart failure	200 (18.8)	85 (20.0)	115 (18.0)	0.424	70 (18.3)	27 (16.6)	43 (19.6)	0.504
Cardiac arrhythmias	141 (13.2)	68 (16.0)	73 (11.4)	0.034	60 (15.7)	27 (16.6)	33 (15.1)	0.776
Valvular disease	79 (7.4)	36 (8.5)	43 (6.7)	0.286	14 (3.7)	5 (3.1)	9 (4.1)	0.784
Pulmonary circulation disorders	111 (10.4)	39 (9.2)	72 (11.2)	0.306	24 (6.3)	17 (10.4)	7 (3.2)	0.005
Hypertension	439 (41.2)	185 (43.5)	254 (39.7)	0.227	204 (53.4)	89 (54.6)	115 (52.5)	0.756
Paralysis	50 (4.7)	13 (3.1)	37 (5.8)	0.053	9 (2.4)	1 (0.6)	8 (3.7)	0.084
Other neurologic disorders	117 (11.0)	40 (9.4)	77 (12.0)	0.194	39 (10.2)	14 (8.6)	25 (11.4)	0.398
Diabetes, uncomplicated	142 (13.3)	54 (12.7)	88 (13.8)	0.646	97 (25.4)	46 (28.2)	51 (23.3)	0.287
Diabetes, complicated	95 (8.9)	46 (10.8)	49 (7.7)	0.080	50 (13.1)	21 (12.9)	29 (13.2)	1.000
Hypothyroidism	25 (2.3)	15 (3.5)	10 (1.6)	0.061	23 (6.0)	16 (9.8)	7 (3.2)	0.009
Renal failure	143 (13.4)	52 (12.2)	91 (14.2)	0.361	86 (22.5)	36 (22.1)	50 (22.8)	0.902
Liver disease	77 (7.2)	20 (4.7)	57 (8.9)	0.011	34 (8.9)	10 (6.1)	24 (11.0)	0.107
Peptic ulcer disease excluding bleeding	15 (1.4)	4 (0.9)	11 (1.7)	0.427	25 (6.5)	11 (6.7)	14 (6.4)	1.000
AIDS	7 (0.7)	4 (0.9)	3 (0.5)	0.446	2 (0.5)	0 (0.0)	2 (0.9)	0.509
Lymphoma	7 (0.7)	3 (0.7)	4 (0.6)	1.000	8 (2.1)	5 (3.1)	3 (1.4)	0.294
Metastatic cancer	50 (4.7)	25 (5.9)	25 (3.9)	0.141	14 (3.7)	6 (3.7)	8 (3.7)	1.000
Solid tumor without metastasis	42 (3.9)	13 (3.1)	29 (4.5)	0.262	38 (9.9)	17 (10.4)	21 (9.6)	0.863
Rheumatoid arthritis/collagen vascular disease	25 (2.3)	17 (4.0)	8 (1.2)	0.006	4 (1.0)	2 (1.2)	2 (0.9)	1.000
Coagulopathy	45 (4.2)	18 (4.2)	27 (4.2)	1.000	9 (2.4)	5 (3.1)	4 (1.8)	0.505
Obesity	73 (6.9)	33 (7.8)	40 (6.2)	0.386	51 (13.4)	31 (19.0)	20 (9.1)	0.006

Continue...

...continuation

Weight loss	49 (4.6)	21 (4.9)	28 (4.4)	0.658	13 (3.4)	5 (3.1)	8 (3.7)	1.000
Fluid and electrolyte disorders	147 (13.8)	57 (13.4)	90 (14.1)	0.786	22 (5.8)	6 (3.7)	16 (7.3)	0.182
Blood loss anemia	43 (4.0)	13 (3.1)	30 (4.7)	0.206	7 (1.8)	2 (1.2)	5 (2.3)	0.704
Deficiency anemia	43 (4.0)	26 (6.1)	17 (2.7)	0.006	20 (5.2)	5 (3.1)	15 (6.8)	0.110
ALcohol abuse	31 (2.9)	2 (0.5)	29 (4.5)	< 0.001	34 (8.9)	6 (3.7)	28 (12.8)	0.002
Drug abuse	11 (1.0)	2 (0.5)	9 (1.4)	0.216	12 (3.1)	5 (3.1)	7 (3.2)	1.000
Psychosis	13 (1.2)	6 (1.4)	7 (1.1)	0.777	11 (2.9)	3 (1.8)	8 (3.7)	0.366
Depression	20 (1.9)	4 (0.9)	16 (2.5)	0.104	39 (10.2)	23 (14.1)	16 (7.3)	0.040
Other	245 (23.0)	96 (22.6)	149 (23.3)	0.824	145 (38.0)	62 (38.0)	83 (37.9)	1.000
None	82 (7.7)	33 (7.8)	49 (7.7)	1.000	6 (1.6)	1 (0.6)	5 (2.3)	0.245

LMIC - low- and middle- income countries; HIC - high–income countries; ED - emergency department; SOFA - Sequential Organ Failure Assessment. Data are median (interquartile range) or n (%). Percentages may not total 100 because of rounding.

Table 1SB - After implementation: baseline characteristics low- and middle-income and high-income countries

	LMIC				HIC			
	Overall, n = 2,168	Female, n = 871 (40%)	Male, n = 1,297 (60%)	p value	Overall, n = 641	Female, n = 298 (46%)	Male, n = 343 (54%)	p value
Age	61.0 [44.8 - 74.2]	61.3 [43.9 - 75.4]	60.9 [45.5 - 73.4]	0.860	65.4 [52.4 - 75.3]	66.6 [54.5 - 76.3]	64.1 [50.4 - 74.3]	0.039
Weight	67.0 [58.0 - 79.0]	60.0 [53.0 - 71.0]	70.0 [63.0 - 80.0]	< 0.001	77.0 [65.0 - 90.0]	70.0 [60.0 - 88.4]	80.0 [70.0 - 93.7]	< 0.001
Height	167.0 [160.0 - 172.0]	160.0 [155.0 - 165.0]	170.0 [167.0 - 175.0]	< 0.001	168.0 [160.0 - 175.0]	160.0 [155.0 - 165.0]	175.0 [170.0 - 180.0]	< 0.001
Hospital admission source				0.257				0.745
Home	1305 (60.2)	538 (61.8)	767 (59.2)		300 (46.9)	146 (49.3)	154 (44.9)	
Nursing home	22 (1.0)	7 (0.8)	15 (1.2)		17 (2.7)	9 (3.0)	8 (2.3)	
ED	587 (27.1)	240 (27.6)	347 (26.8)		204 (31.9)	88 (29.7)	116 (33.8)	
Outside hospital ED	203 (9.4)	69 (7.9)	134 (10.3)		77 (12.1)	34 (11.5)	43 (12.5)	
Other	50 (2.3)	17 (2.0)	33 (2.5)		41 (6.4)	19 (6.4)	22 (6.4)	
Life support limitations	88 (4.1)	42 (4.9)	46 (3.6)	0.150	197 (31.0)	103 (35.0)	94 (27.5)	0.048
SOFA	6.0 [3.0 - 9.0]	5.0 [3.0 - 9.0]	6.0 [4.0 - 9.0]	0.010	7.0 [4.0 - 10.0]	7.0 [4.0 - 10.0]	7.0 [5.0 - 10.0]	0.297
Mechanical ventilation	1305 (60.9)	508 (58.9)	797 (62.2)	0.137	375 (58.8)	165 (55.6)	210 (61.6)	0.126
Comorbidities								
Congestive heart failure	232 (10.7)	94 (10.8)	138 (10.6)	0.944	144 (22.5)	68 (22.8)	76 (22.2)	0.850
Cardiac arrhythmias	187 (8.6)	76 (8.7)	111 (8.6)	0.938	109 (17.0)	50 (16.8)	59 (17.2)	0.916
Valvular disease	71 (3.3)	33 (3.8)	38 (2.9)	0.271	39 (6.1)	21 (7.0)	18 (5.2)	0.408
Pulmonary circulation disorders	132 (6.1)	48 (5.5)	84 (6.5)	0.410	61 (9.5)	36 (12.1)	25 (7.3)	0.043
Hypertension	918 (42.3)	368 (42.3)	550 (42.4)	0.965	286 (44.6)	154 (51.7)	132 (38.5)	0.001
Paralysis	52 (2.4)	27 (3.1)	25 (1.9)	0.087	16 (2.5)	7 (2.3)	9 (2.6)	1.000
Other neurologic disorders	137 (6.3)	44 (5.1)	93 (7.2)	0.048	69 (10.8)	38 (12.8)	31 (9.0)	0.160

Continue...

...continuation

Diabetes, uncomplicated	292 (13.5)	127 (14.6)	165 (12.7)	0.223	103 (16.1)	54 (18.1)	49 (14.3)	0.197
Diabetes, complicated	205 (9.5)	78 (9.0)	127 (9.8)	0.550	113 (17.6)	61 (20.5)	52 (15.2)	0.096
Hypothyroidism	59 (2.7)	36 (4.1)	23 (1.8)	0.001	52 (8.1)	35 (11.7)	17 (5.0)	0.002
Renal failure	238 (11.0)	90 (10.3)	148 (11.4)	0.442	121 (18.9)	63 (21.1)	58 (16.9)	0.189
Liver disease	103 (4.8)	45 (5.2)	58 (4.5)	0.472	39 (6.1)	11 (3.7)	28 (8.2)	0.020
Peptic ulcer disease excluding bleeding	38 (1.8)	16 (1.8)	22 (1.7)	0.868	10 (1.6)	5 (1.7)	5 (1.5)	1.000
AIDS	12 (0.6)	1 (0.1)	11 (0.8)	0.034	0 (0.0)	0 (0.0)	0 (0.0)	NA
Lymphoma	41 (1.9)	18 (2.1)	23 (1.8)	0.633	8 (1.2)	1 (0.3)	7 (2.0)	0.074
Metastatic cancer	117 (5.4)	43 (4.9)	74 (5.7)	0.498	23 (3.6)	10 (3.4)	13 (3.8)	0.834
Solid tumor without metastasis	158 (7.3)	71 (8.2)	87 (6.7)	0.207	38 (5.9)	20 (6.7)	18 (5.2)	0.503
Rheumatoid arthritis/collagen vascular disease	45 (2.1)	31 (3.6)	14 (1.1)	< 0.001	10 (1.6)	8 (2.7)	2 (0.6)	0.051
Coagulopathy	58 (2.7)	23 (2.6)	35 (2.7)	1.000	23 (3.6)	11 (3.7)	12 (3.5)	1.000
Obesity	58 (2.7)	34 (3.9)	24 (1.9)	0.004	65 (10.1)	38 (12.8)	27 (7.9)	0.049
Weight loss	69 (3.2)	30 (3.4)	39 (3.0)	0.618	10 (1.6)	6 (2.0)	4 (1.2)	0.526
Fluid and electrolyte disorders	195 (9.0)	75 (8.6)	120 (9.3)	0.646	43 (6.7)	23 (7.7)	20 (5.8)	0.348
Blood loss anemia	81 (3.7)	33 (3.8)	48 (3.7)	0.909	21 (3.3)	12 (4.0)	9 (2.6)	0.377
Deficiency anemia	35 (1.6)	19 (2.2)	16 (1.2)	0.116	26 (4.1)	17 (5.7)	9 (2.6)	0.069
ALcohol abuse	20 (0.9)	3 (0.3)	17 (1.3)	0.022	70 (10.9)	14 (4.7)	56 (16.3)	< 0.001
Drug abuse	15 (0.7)	3 (0.3)	12 (0.9)	0.122	31 (4.8)	10 (3.4)	21 (6.1)	0.139
Psychosis	26 (1.2)	12 (1.4)	14 (1.1)	0.551	14 (2.2)	8 (2.7)	6 (1.7)	0.433
Depression	29 (1.3)	12 (1.4)	17 (1.3)	1.000	46 (7.2)	29 (9.7)	17 (5.0)	0.022
Other	478 (22.0)	218 (25.0)	260 (20.0)	0.007	225 (35.1)	101 (33.9)	124 (36.2)	0.562
None	323 (14.9)	119 (13.7)	204 (15.7)	0.197	39 (6.1)	16 (5.4)	23 (6.7)	0.512

LMIC - low- and middle- income countries; HIC - high–income countries; ED - emergency department; SOFA - Sequential Organ Failure Assessment. Data are median (interquartile range) or n (%). Percentages may not total 100 because of rounding.

Table 2SA - Before implementation: the incidence rates of omissions in daily care processes

Total = 1,364 Observed event	Male, n = 811 Incidence rate (95%CI)	Observation days, n	Observed events, n	Patients, n	Female, n = 553 Incidence rate (95%CI)	Observation days, n	Observed events, n	Patients, n	Adjusted for center effects incidence rate ratio	p value
	**Per 1,000 ventilator days**				**Per 1,000 ventilator days**					
No deep vein thrombosis prophylaxis	273 (249 - 300)	1623	443	533	226 (199 - 258)	1008	228	338	1.00 (0.85 - 1.18)	0.99
No peptic ulcer prophylaxis	42.5 (33.6 - 53.8)	1623	69	533	64.5 (50.6 - 82.2)	1008	65	338	1.44 (1.02 - 2.02)	0.04
No documented assessment of spontaneous breathing trial	579 (543 - 617)	1623	939	533	507 (465 - 553)	1008	511	338	0.92 (0.82 - 1.03)	0.13
No documented family conference/ discussion	417 (386 - 449)	1623	676	533	419 (381 - 461)	1008	422	338	1.06 (0.93 - 1.20)	0.38
No daily oral care	24.6 (18.1 - 33.6)	1623	40	533	46.6 (35.0 - 62.1)	1008	47	338	1.64 (1.06 - 2.52)	0.03
No head of bed elevation at 30	22.2 (16.0 - 30.8)	1623	36	533	25.8 (17.6 - 37.9)	1008	26	338	1.25 (0.74 - 2.11)	0.41
	**Per 1,000 CVC days**				**Per 1,000 CVC days**					
No documented assessment for CVC removal	667 (632 - 705)	1906	1272	580	607 (564 - 653)	1186	720	361	0.97 (0.89 - 1.07)	0.57
	**Per 1,000 urinary catheter days**				**Per 1,000 urinary catheter days**					
No documented assessment for urinary catheter removal	690 (658 - 724)	2432	1679	743	653 (615 - 694)	1593	1041	498	0.98 (0.90 - 1.06)	0.55
	**Per 1,000 days of use**				**Per 1,000 days of use**					
No documented assessment to continue or discontinue current antimicrobials	339 (316 - 363)	2384	808	722	318 (292 - 347)	1586	505	497	0.96 (0.86 - 1.07)	0.42
No documented assessment to continue or discontinue current sedation	359 (327 - 387)	1479	526	514	349 (311 - 393)	796	278	302	1.02 (0.88 - 1.18)	0.82

95%CI - 95% confidence interval; CVC - central venous catheter.

Table 2SB - After implementation: the incidence rates of omissions in daily care processes

Total = 2,663<br>Observed event	Male, n = 1,557<br>Incidence rate (95%CI)	Observation days, n	Observed events, n	Patients, n	Female, n = 1,106<br>Incidence rate (95%CI)	Observation days, n	Observed events, n	Patients, n	Adjusted for center effects incidence rate ratio	p value
	**Per 1,000 ventilator days**				**Per 1,000 ventilator days**					
No deep vein thrombosis prophylaxis	192 (177 - 208)	3,012	577	1,013	182 (164 - 203)	1,924	351	660	0.96 (0.84 - 1.10)	0.53
No peptic ulcer prophylaxis	28.9 (23.4 - 35.6)	3,012	87	1,013	28.6 (21.9 - 37.2)	1,924	55	660	0.94 (0.67 - 1.32)	0.71
No documented assessment of spontaneous breathing trial	451 (428 - 476)	3,012	1,359	1,013	415 (387 - 445)	1,924	798	660	0.94 (0.86 - 1.03)	0.17
No documented family conference/ discussion	353 (333 - 375)	3,012	1,064	1,013	385 (358 - 414)	1,924	741	660	1.05 (0.95 - 1.15)	0.35
No daily oral care	28.6 (23.1 - 35.3)	3,012	86	1,013	41.6 (33.4 - 51.8)	1,924	80	660	1.30 (0.96 - 1.77)	0.10
No head of bed elevation at 30	29.9 (24.3 - 36.7)	3,012	90	1,013	38.5 (30.6 - 48.3)	1,924	74	660	1.42 (1.04 - 1.94)	0.03
	**Per 1,000 CVC days**				**Per 1,000 CVC days**					
No documented assessment for CVC removal	565 (541 - 591)	3,463	1,958	1,092	553 (522 - 585)	2,177	1,203	709	1.03 (0.95 - 1.10)	0.48
	**Per 1,000 urinary catheter days**				**Per 1,000 urinary catheter days**					
No documented assessment for urinary catheter removal	571 (550 - 594)	4,637	2,650	1,462	549 (524 - 576)	3,092	1,699	1,024	1.02 (0.96 - 1.08)	0.63
	**Per 1,000 days of use**				**Per 1,000 days of use**					
No documented assessment to continue or discontinue current antimicrobials	216 (203 - 231)	4,311	933	1,392	195 (180 - 212)	2,930	572	980	0.97 (0.87 - 1.07)	0.51
No documented assessment to continue or discontinue current sedation	200 (184 -218)	2,665	533	996	210 (189 - 233)	1,621	340	639	1.05 (0.91 - 1.20)	0.50

95%CI - 95% confidence interval; CVC - central venous catheter.

Table 3SA - Before implementation low- and middle- income countries: the incidence rates of omissions in daily care processes

Total = 1,008 Observed event	Male, n = 610 Incidence rate (95%CI)	Observation days, n	Observed events, n	Patients, n	Female, n = 398 Incidence rate (95%CI)	Observation days, n	Observed events, n	Patients, n	Adjusted for center effects incidence rate ratio	p value
	**Per 1,000 ventilator days**				**Per 1,000 ventilator days**					
No deep vein thrombosis prophylaxis	331 (301 - 363)	1,301	430	421	279 (245 - 319)	773	216	252	0.98 (0.83 - 1.16)	0.85
No peptic ulcer prophylaxis	52.3 (41.2 - 66.3)	1,301	68	421	80.2 (62.5 - 103)	773	62	252	1.37 (0.97 - 1.93)	0.08
No documented assessment of spontaneous breathing trial	609 (568 - 653)	1,301	792	421	521 (473 - 575)	773	403	252	0.89 (0.78 - 1.00)	0.05
No documented family conference/ discussion	462 (426 - 500)	1,301	601	421	472 (426 - 523)	773	365	252	1.04 (0.91 - 1.18)	0.6
No daily oral care	26.1 (18.7 - 36.6)	1,301	34	421	40.1 (28.2 - 57.0)	773	31	252	1.30 (0.79 - 2.15)	0.3
No head of bed elevation at 30	23.8 (16.8 - 33.9)	1,301	31	421	28.5 (18.7 - 43.2)	773	22	252	1.28 (0.72 - 2.26)	0.4
	**Per 1,000 CVC days**				**Per 1,000 CVC days**					
No documented assessment for CVC removal	682 (641 - 725)	1,495	1,019	440	629 (579 - 683)	892	561	262	0.98 (0.88 - 1.08)	0.67
	**Per 1,000 urinary catheter days**				**Per 1,000 urinary catheter days**					
No documented assessment for urinary catheter removal	684 (648 - 722)	1,938	1,326	575	647 (604 - 693)	1,249	808	376	0.96 (0.88 - 1.05)	0.39
	**Per 1,000 days of use**				**Per 1,000 days of use**					
No documented assessment to continue or discontinue current antimicrobials	350 (325 - 378)	1,907	668	559	351 (320 - 386)	1,239	435	374	0.98 (0.87 - 1.11)	0.78
No documented assessment to continue or discontinue current sedation	368 (334 - 405)	1,123	413	384	360 (315 - 412)	586	211	216	0.98 (0.83 - 1.16)	0.83

95%CI - 95% confidence interval CVC - central venous catheter.

Table 3SB - After implementation low- and middle- income countries: the incidence rates of omissions in daily care processes

Total = 2,102 Observed event	Male, n = 1,257 Incidence rate (95%CI)	Observation days, n	Observed events, n	Patients, n	Female, n = 845 Incidence rate (95%CI)	Observation days, n	Observed events, n	Patients, n	Adjusted for center effects incidence rate ratio	p value
	**Per 1,000 ventilator days**				**Per 1,000 ventilator days**					
No deep vein thrombosis prophylaxis	218 (200 - 237)	2,463	537	821	217 (195 - 242)	1,482	322	507	1.00 (0.87 - 1.15)	0.96
No peptic ulcer prophylaxis	32.1 (25.7 - 40.0)	2,463	79	821	35.1 (26.7 - 46.0)	1,482	52	507	1.04 (0.73 - 1.48)	0.84
No documented assessment of spontaneous breathing trial	497 (469 - 525)	2,463	1,223	821	462 (428 - 497)	1,482	684	507	0.94 (0.85 - 1.03)	0.18
No documented family conference/ discussion	338 (316 - 362)	2,463	833	821	370 (340 - 402)	1,482	548	507	1.05 (0.94 - 1.17)	0.43
No daily oral care	24.0 (18.6 - 30.9)	2,463	59	821	39.8 (30.8 - 51.4)	1,482	59	507	1.65 (1.14 - 2.38)	0.008
No head of bed elevation at 30	34.9 (28.3 - 43.1)	2,463	86	821	48.6 (38.6 - 61.2)	1,482	72	507	1.46 (1.07 - 2.00)	0.02
	**Per 1,000 CVC days**				**Per 1,000 CVC days**					
No documented assessment for CVC removal	612 (583 - 642)	2,795	1,710	870	593 (557 - 631)	1,655	981	539	1.03 (0.95 - 1.11)	0.50
	**Per 1,000 urinary catheter days**				**Per 1,000 urinary catheter days**					
No documented assessment for urinary catheter removal	612 (588 - 637)	3,887	2,380	1,207	596 (566 - 627)	2,469	1,471	812	1.02 (0.95 - 1.09)	0.57
	**Per 1,000 days of use**				**Per 1,000 days of use**					
No documented assessment to continue or discontinue current antimicrobials	230 (214 - 246)	3,552	816	1,130	213 (195 - 233)	2,277	485	751	0.96 (0.86 - 1.07)	0.47
No documented assessment to continue or discontinue current sedation	210 (191 - 230)	2,122	445	792	218 (193 - 246)	1,210	264	483	1.03 (0.89 - 1.21)	0.68

95%CI - 95% confidence interval; CVC - central venous catheter.

Table 4SA - Before implementation high–income countries: the incidence rates of omissions in daily care processes

Total = 356 Observed event	Male, n = 201 Incidence rate (95%CI)	Observation days, n	Observed events, n	Patients, n	Female, n = 155 Incidence rate (95%CI)	Observation days, n	Observed events, n	Patients, n	Adjusted for center effects incidence rate ratio	p value
	**Per 1,000 ventilator days**				**Per 1,000 ventilator days**					
No deep vein thrombosis prophylaxis	40.4 (23.4 - 69.5)	322	13	112	51.1 (29.0 - 89.9)	235	12	86	1.20 (0.54 - 2.60	0.65
No peptic ulcer prophylaxis	3.11 (0.44 - 22.0)	322	1	112	12.8 (4.12 - 39.6)	235	3	86	4.16 (0.42 - 41.0)	0.19
No documented assessment of spontaneous breathing trial	457 (388 - 537)	322	147	112	460 (381 - 555)	235	108	86	1.08 (0.84 - 1.40)	0.54
No documented family conference/ discussion	233 (186 - 292)	322	75	112	243 (187 - 314)	235	57	86	1.24 (0.87 - 1.77)	0.24
No daily oral care	18.6 (8.37 - 41.5)	322	6	112	68.1 (41.7 - 111.1)	235	16	86	3.68 (1.42 - 9.55)	0.004
No head of bed elevation at 30	15.5 (6.46 - 37.3)	322	5	112	17.0 (6.39 - 45.4)	235	4	86	1.10 (0.29 - 4.16)	0.89
	**Per 1,000 CVC days**				**Per 1,000 CVC days**					
No documented assessment for CVC removal	616 (544 - 696)	411	253	140	541 (463 - 632)	294	159	99	0.95 (0.78 - 1.16)	0.61
	**Per 1,000 urinary catheter days**				**Per 1,000 urinary catheter days**					
No documented assessment for urinary catheter removal	715 (644 - 793)	494	353	168	677 (596 - 770)	344	233	122	1.02 (0.87 - 1.21)	0.78
	**Per 1,000 days of use**				**Per 1,000 days of use**					
No documented assessment to continue or discontinue current antimicrobials	294 (249 - 346)	477	140	163	202 (160 - 255)	347	70	123	0.79 (0.60 - 1.06)	0.11
No documented assessment to continue or discontinue current sedation	317 (264 - 382)	356	113	130	319 (251- 405)	210	67	86	1.11 (0.82 - 1.51)	0.49

95%CI - 95% confidence interval; CVC - central venous catheter.

Table 4SB - After implementation high-income countries: the incidence rates of omissions in daily care processes

Total = 561<br>Observed event	Male, n = 300<br>Incidence rate<br>(95%CI)	Observation days, n	Observed events, n	Patients, n	Female, n = 261<br>Incidence rate<br>(95%CI)	Observation days, n	Observed events, n	Patients, n	Adjusted for center effects incidence rate ratio	p value
	**Per 1,000 ventilator days**				**Per 1,000 ventilator days**					
No deep vein thrombosis prophylaxis	72.9 (53.4 - 99.3)	549	40	192	65.6 (45.6 - 94.4)	442	29	153	0.64 (0.39 - 1.04)	0.07
No peptic ulcer prophylaxis	14.6 (7.29 - 29.1)	549	8	192	6.79 (2.19 - 21.0)	442	3	153	0.35 (0.09 - 1.33)	0.10
No documented assessment of spontaneous breathing trial	248 (209 - 293)	549	136	192	258 (215- 310)	442	114	153	0.97 (0.75 - 1.25)	0.83
No documented family conference/ discussion	421 (370 - 479)	549	231	192	437 (379 - 503)	442	193	153	1.01 (0.83 - 1.23)	0.89
No daily oral care	49.2 (33.7 - 71.7)	549	27	192	47.5 (31.0 - 72.9)	442	21	153	0.73 (0.41 - 1.29)	0.29
No head of bed elevation at 30	7.29 (2.73 - 19.4)	549	4	192	4.52 (1.13 - 18.1)	442	2	153	0.72 (0.12 - 4.16)	0.71
	**Per 1,000 CVC days**				**Per 1,000 CVC days**					
No documented assessment for CVC removal	371 (328 - 420)	668	248	222	425 (373 - 485)	522	222	170	0.98 (0.82 - 1.18)	0.85
	**Per 1,000 urinary catheter days**				**Per 1,000 urinary catheter days**					
No documented assessment for urinary catheter removal	360 (320 - 406)	750	270	255	366 (321 - 417)	623	228	212	0.96 (0.80 - 1.14)	0.65
	**Per 1,000 days of use**				**Per 1,000 days of use**					
No documented assessment to continue or discontinue current antimicrobials	154 (129 - 185)	759	117	262	133 (108 - 164)	653	87	229	0.94 (0.71 - 1.24)	0.67
No documented assessment to continue or discontinue current sedation	162 (132 - 200)	543	88	204	185 (148 - 232)	411	76	156	0.99 (0.73 - 1.35)	0.95

95%CI - 95% confidence interval; CVC - central venous catheter.

Table 5S - Low- and middle- income countries: mortality before and after checklist of early recognition and treatment of acute illness and injury implementation

Before								
**Total = 1,065**	**Male, n = 640 Mortality (%)**	**Death**	**Patient, n**	**Female, n = 425 Mortality (%)**	**Death**	**Patient, n**	**Effect measure (95%CI) Adjusted OR**	**p value**
ICU mortality	31.1 (27.6 - 34.8)	197	634	32.5 (28.2 - 37.2)	135	415	1.06 (0.80 - 1.39)	0.69
Hospital mortality	35.7 (32.1 - 39.5)	225	630	37.2 (32.7 - 42.0)	153	411	1.04 (0.79 - 1.36)	0.8
28 d mortality	39.2 (35.4 - 43.1)	244	623	40.8 (36.1 - 45.7)	164	402	1.11 (0.85 - 1.45)	0.45
**After**								
**Total = 2,167**	**Male, n = 1,296 Mortality (%)**	**Death**	**Patient, n**	**Female, n = 871 Mortality (%)**	**Death**	**Patient, n**	**Effect measure (95%CI) Adjusted OR**	**p value**
ICU mortality	23.8 (21.6 - 26.2)	308	1293	22.9 (20.2 - 25.8)	199	869	0.94 (0.75 - 1.17)	0.57
Hospital mortality	28.8 (26.4 - 31.4)	372	1290	28.1 (25.2 - 31.2)	244	869	0.94 (0.76 - 1.15)	0.53
28 d mortality	31.5 (29.0 - 34.1)	406	1289	30.1 (27.2 - 33.2)	262	870	0.92 (0.75 - 1.12)	0.41

95%CI - 95% confidence interval; OR - odds ratio; ICU - intensive care unit.

Table 6S - High-income countries: mortality before and after checklist of early recognition and treatment of acute illness and injury implementation

Before								
**Total = 382**	**Male, n = 219 Mortality (%)**	**Death**	**Patient, n**	**Female, n = 163 Mortality (%)**	**Death**	**Patient, n**	**Effect measure (95% CI) Adjusted OR**	**p value**
ICU mortality	21.3 (16.2 - 27.5)	43	202	19.6 (14.2 - 26.6)	31	158	0.97 (0.57 - 1.67)	0.92
Hospital mortality	29.6 (23.7 - 36.4)	59	199	25.6 (19.4 - 33.1)	40	156	0.86 (0.53 - 1.41)	0.55
28 d mortality	28.6 (22.6 - 35.4)	54	189	27.5 (21.0 - 35.1)	42	153	1.01 (0.61 - 1.66)	0.98
**After**								
**Total = 641**	**Male, n = 343 Mortality (%)**	**Death**	**Patient, n**	**Female, n = 298 Mortality (%)**	**Death**	**Patient, n**	**Effect measure (95%CI) Adjusted OR**	**p value**
ICU mortality	24.4 (20.0 - 29.4)	78	320	22.5 (18.0 - 27.8)	63	280	0.82 (0.54 - 1.24)	0.34
Hospital mortality	30.7 (25.9 - 36.0)	98	319	28.3 (23.3 - 33.9)	79	279	0.83 (0.56 - 1.22)	0.34
28 d mortality	32.0 (27.0 - 37.5)	98	306	29.7 (24.5 - 35.5)	79	266	0.79 (0.53 - 1.18)	0.25

95%CI - 95% confidence interval; OR - odds ratio; ICU - intensive care unit.

Table 7S - Low- and middle- income countries: intensive care unit. and hospital length of stay before and after checklist of early recognition and treatment of acute illness and injury implementation

Before				
**Total = 1,065**	**Male, n = 640 Geometric mean (SD), d**	**Female, n = 425 Geometric mean (SD), d**	**Effect measure (95%CI) Adjusted ratio of geometric means**	**p value**
ICU length of stay	7.41 (2.97)	7.25 (3.08)	0.99 (0.87 - 1.13)	0.91
Hospital Length of stay	16.1 (3.03)	16.0 (2.94)	1.00 (0.87 - 1.14)	0.94
**After**				
**Total = 2,167**	**Male, n = 1,296 Geometric mean (SD), d**	**Female, n = 871 Geometric mean (SD), d**	**Effect measure (95%CI) Adjusted ratio of geometric means**	**p value**
ICU length of stay	6.54 (2.87)	5.69 (2.70)	0.89 (0.82 - 0.98)	0.01
Hospital Length of stay	16.1 (2.70)	15.0 (2.65)	0.97 (0.89 - 1.05)	0.42

SD - standard deviation; 95%CI - 95% confidence interval; ICU - intensive care unit.

Table 8S - High–income countries: intensive care unit and hospital length of stay before and after checklist of early recognition and treatment of acute illness and injury implementation

**Before**				
**Total = 382**	**Male, n= 219 Geometric mean (SD), d**	**Female, n = 163 Geometric mean (SD), d**	**Effect measure (95%CI) Adjusted ratio of geometric means**	**p value**
ICU length of stay	4.69 (2.78)	4.45 (2.44)	0.95 (0.80 - 1.14)	0.60
Hospital Length of stay	12.6 (3.28)	11.1 (3.01)	0.86 (0.70 - 1.07)	0.17
**After**				
**Total = 641**	**Male, n= 343 Geometric mean (SD), d**	**Female, n = 298 Geometric mean (SD), d**	**Effect measure (95%CI) Adjusted ratio of geometric means**	**p value**
ICU length of stay	4.70 (2.79)	4.40 (2.82)	0.97 (0.83 - 1.14)	0.71
Hospital Length of stay	11.2 (3.12)	11.4 (3.32)	1.09 (0.91 - 1.29)	0.35

SD - standard deviation; 95%CI - 95% confidence interval; ICU - intensive care unit.

Table 9SA - Males: the incidence rates of omissions in daily care processes

**Total = 2368 Observed event**	**Before, n = 811 Incidence rate (95%CI)**	**Observation days, n**	**Observed events, n**	**Patients, n**	**After, n = 1,557 Incidence rate (95%CI)**	**Observation days, n**	**Observed events, n**	**Patients, n**	**Adjusted for center effects incidence rate ratio**	**p value**
	**Per 1,000 ventilator days**				**Per 1,000 ventilator days**					
No deep vein thrombosis prophylaxis	273 (249 - 300)	1,623	443	533	192 (177 - 208)	3,012	577	1,013	0.71 (0.63 - 0.81)	<.0.001
No peptic ulcer prophylaxis	42.5 (33.6 - 53.8)	1,623	69	533	28.9 (23.4 - 35.6)	3,012	87	1,013	0.60 (0.44 - 0.83)	0.002
No documented assessment of spontaneous breathing trial	579 (543 - 617)	1,623	939	533	451 (428 - 476)	3,012	1359	1,013	0.78 (0.72 - 0.85)	< 0.001
No documented family conference/ discussion	417 (386 - 449)	1,623	676	533	353 (333 - 375)	3,012	1,064	1,013	0.87 (0.78 - 0.95)	0.004
No daily oral care	24.6 (18.1 - 33.6)	1,623	40	533	28.6 (23.1 - 35.3)	3,012	86	1,013	1.17 (0.80 - 1.71)	0.42
No head of bed elevation at 30	22.2 (16.0 - 30.8)	1,623	36	533	29.9 (24.3 - 36.7)	3,012	90	1,013	1.40 (0.95 - 2.07)	0.084
	**Per 1,000 CVC days**				**Per 1,000 CVC days**					
No documented assessment for CVC removal	667 (632 - 705)	1,906	1,272	580	565 (541 - 591)	3,463	1,958	1,092	0.81 (0.75 - 0.87)	< 0.001
	**Per 1,000 urinary catheter days**				**Per 1,000 urinary catheter days**					
No documented assessment for urinary catheter removal	690 (658 - 724)	2,432	1,679	743	571 (550 - 594)	4,637	2,650	1,462	0.81 (0.76 - 0.86)	<.0.001***
	**Per 1,000 days of use**				**Per 1,000 days of use**					
No documented assessment to continue or discontinue current antimicrobials	339 (316 - 363)	2,384	808	722	216 (203 - 231)	43,11	933	1,392	0.63 (0.58 - 0.70)	<.0,001***
No documented assessment to continue or discontinue current sedation	359 (327 - 387)	1,479	526	514	200 (184 - 218)	2,665	533	996	0.56 (0.53 - 0.67)	< 0.001***

95%CI - 95% confidence interval; CVC - central venous catheter.

Table 9SB - Females: the incidence rates of omissions in daily care processes

Total = 1,659 Observed event	Before, n = 553 Incidence rate (95%CI)	Observation days, n	Observed events, n	Patients, n	After, n = 1106 Incidence rate (95%CI)	Observation days, n	Observed events, n	Patients, n	Adjusted for center effects incidence rate ratio	p value
	**Per 1,000 ventilator days**				**Per 1,000 ventilator days**					
No deep vein thrombosis prophylaxis	226 (199 - 258)	1,008	228	338	182 (164 - 203)	1,924	351	660	0.75 (0.64 - 0.89)	0.001
No peptic ulcer prophylaxis	64.5 (50.6 - 82.2)	1,008	65	338	28.6 (21.9 - 37.2)	1,924	55	660	0.48 (0.33 - 0.69)	< 0.001
No documented assessment of spontaneous breathing trial	507 (465 - 553)	1,008	511	338	415 (387 - 445)	1,924	798	660	0.83 (0.74 - 0.93)	0.001
No documented family conference/ discussion	419 (381 - 461)	1,008	422	338	385 (358 - 414)	1,924	741	660	0.87 (0.78 - 0.94)	0.03
No daily oral care	46.6 (35.0 - 62.1)	1,008	47	338	41.6 (33.4 - 51.8)	1,924	80	660	0.86 (0.59 - 1.25)	0.43
No head of bed elevation at 30	25.8 (17.6 - 37.9)	1,008	26	338	38.5 (30.6 - 48.3)	1,924	74	660	1.38 (0.88 - 2.17)	0.16
	**Per 1,000 CVC days**				**Per 1,000 CVC days**					
No documented assessment for CVC removal	607 (564 - 653)	1,186	720	361	553 (522 - 585)	2,177	1,203	709	0.91 (0.83 - 0.99)	0.04
	**Per 1,000 urinary catheter days**				**Per 1,000 urinary catheter days**					
No documented assessment for urinary catheter removal	653 (615 - 694)	1,593	1,041	498	549 (524 - 576)	3,092	1,699	1,024	0.88 (0.81 - 0.95)	< 0.001
	**Per 1,000 days of use**				**Per 1,000 days of use**					
No documented assessment to continue or discontinue current antimicrobials	318 (292 - 347)	1,586	505	497	195 (180 - 212)	2,930	572	980	0.67 (0.60 - 0.76)	< 0.001
No documented assessment to continue or discontinue current sedation	349 (311 - 393)	796	278	302	210 (189 - 233)	1,621	340	639	0.65 (0.56 - 0.76)	< 0.001

95%CI - 95% confidence interval; CVC - central venous catheter.

Table 10SA - Males: the incidence rates of omissions in daily care processes low- and middle- income countries

Total = 1,867 Observed event	Before, n = 610 Incidence rate (95%CI)	Observation days, n	Observed events, n	Patients, n	After, n = 1,257 Incidence rate (95%CI)	Observation days, n	Observed events, n	Patients, n	Adjusted for center effects incidence rate ratio	p value
	**Per 1,000 ventilator days**				**Per 1,000 ventilator days**					
No deep vein thrombosis prophylaxis	331 (301 - 363)	1,301	430	421	218 (200 - 237)	2,463	537	821	0.68 (0.59 - 0.77)	< 0.001*
No peptic ulcer prophylaxis	52.3 (41.2 - 66.3)	1,301	68	421	32.1 (25.7 - 40.0)	2,463	79	821	0.54 (0.34 - 0.75)	< 0.001
No documented assessment of spontaneous breathing trial	609 (568 - 653)	1,301	792	421	497 (469 - 525)	2,463	1,223	821	0.82 (0.75 - 0.90)	< 0.001
No documented family conference/ discussion	462 (426 - 500)	1,301	601	421	338 (316 - 362)	2,463	833	821	0.75 (0.68 - 0.84)	< 0.001
No daily oral care	26.1 (18.7 - 36.6)	1,301	34	421	24.0 (18.6 - 30.9)	2,463	59	821	0.87 (0.57 - 1.34)	0.54
No head of bed elevation at 30	23.8 (16.8 - 33.9)	1,301	31	421	34.9 (28.3 - 43.1)	2,463	86	821	1.55 (1.03 - 2.32)	0.03
	**Per 1,000 CVC days**				**Per 1,000 CVC days**					
No documented assessment for CVC removal	682 (641 - 725)	1,495	1,019	440	612 (583 - 642)	2,795	1,710	870	0.84 (0.78 - 0.91)	< 0.001
	**Per 1,000 urinary catheter days**				**Per 1,000 urinary catheter days**					
No documented assessment for urinary catheter removal	684 (648 - 722)	1,938	1,326	575	612 (588 - 637)	3,887	2,380	1,207	0.87 (0.81 - 0.93)	< 0.001
	**Per 1,000 days of use**				**Per 1,000 days of use**					
No documented assessment to continue or discontinue current antimicrobials	350 (325 - 378)	1,907	668	559	230 (214 - 246)	3,552	816	1,130	0.63 (0.57 - 0.70)	< 0.001
No documented assessment to continue or discontinue current sedation	368 (334 - 405)	1,123	413	384	210 (191 - 230)	2,122	445	792	0.59 (0.52 - 0.68)	< 0.001

95%CI - 95% confidence interval; CVC - central venous catheter.

Table 10SB - Females: the incidence rates of omissions in daily care processes low- and middle- income countries

Total = 1243<br>Observed event	Before, n = 398<br>Incidence rate (95%CI)	Observation days, n	Observed events, n	Patients, n	After, n = 845<br>Incidence rate (95%CI)	Observation days, n	Observed events, n	Patients, n	Adjusted for center effects incidence rate ratio	p value
	**Per 1,000 ventilator days**				**Per 1,000 ventilator days**					
No deep vein thrombosis prophylaxis	279 (245 - 319)	773	216	252	217 (195 - 242)	1,482	322	507	0.75 (0.63 - 0.89)	0.001
No peptic ulcer prophylaxis	80.2 (62.5 - 103)	773	62	252	35.1 (26.7 - 46.0)	1,482	52	507	0.49 (0.34 - 0.72)	< 0.001
No documented assessment of spontaneous breathing trial	521 (473 - 575)	773	403	252	462 (428 - 497)	1,482	684	507	0.91 (0.80 - 1.03)	0.12
No documented family conference/ discussion	472 (426 - 523)	773	365	252	370 (340 - 402)	1,482	548	507	0.75 (0.65 - 0.85)	< 0.001
No daily oral care	40.1 (28.2 - 57.0)	773	31	252	39.8 (30.8 - 51.4)	1,482	59	507	0.93 (0.59 - 1.45)	0.74
No head of bed elevation at 30	28.5 (18.7 - 43.2)	773	22	252	48.6 (38.6 - 61.2)	1,482	72	507	1.63 (1.01 - 2.65)	0.04
	**Per 1,000 CVC days**				**Per 1,000 CVC days**					
No documented assessment for CVC removal	629 (579 - 683)	892	561	262	593 (557 - 631)	1,655	981	539	0.97 (0.87 - 1.80)	0.55
	**Per 1,000 urinary catheter days**				**Per 1,000 urinary catheter days**					
No documented assessment for urinary catheter removal	647 (604 - 693)	1,249	808	376	596 (566 - 627)	2,469	1,471	812	0.97 (0.89 - 1.06)	0.5
	**Per 1,000 days of use**				**Per 1,000 days of use**					
No documented assessment to continue or discontinue current antimicrobials	351 (320 - 386)	1,239	435	374	213 (195 - 233)	2,277	485	751	0.66 (0.58 - 0.76)	< 0.001
No documented assessment to continue or discontinue current sedation	360 (315 - 412)	586	211	216	218 (193 - 246)	1,210	264	483	0.69 (0.57 - 0.82)	< 0.001

95%CI - 95% confidence interval; CVC - central venous catheter.

Table 11SA - Males: the incidence rates of omissions in daily care processes high–income countries

Total = 501<br>Observed event	Before, n = 201<br>Incidence rate (95%CI)	Observation days, n	Observed events, n	Patients, n	After, n = 300<br>Incidence rate (95%CI)	Observation days, n	Observed events, n	Patients, n	Adjusted for center effects incidence rate ratio	p value
	**Per 1,000 ventilator days**				**Per 1,000 ventilator days**					
No deep vein thrombosis prophylaxis	40.4 (23.4 - 69.5)	322	13	112	72.9 (53.4 - 99.3)	549	40	192	1.78 (0.95 - 3.33)	0.06
No peptic ulcer prophylaxis	3.11 (0.44 - 22.0)	322	1	112	14.6 (7.29 - 29.1)	549	8	192	4.42 (0.55 - 35.3)	0.09
No documented assessment of spontaneous breathing trial	457 (388 - 537)	322	147	112	248 (209 - 293)	549	136	192	0.55 (0.44 - 0.70)	< 0.001
No documented family conference/ discussion	233 (186 - 292)	322	75	112	421 (370 - 479)	549	231	192	1.85 (1.42 - 2.41)	< 0.001
No daily oral care	18.6 (8.37 - 41.5)	322	6	112	49.2 (33.7 - 71.7)	549	27	192	3.11 (1.29 - 7.50)	0.007
No head of bed elevation at 30	15.5 (6.46 - 37.3)	322	5	112	7.29 (2.73 - 19.4)	549	4	192	0.47 (0.13 - 1.75)	0.26
	**Per 1,000 CVC days**				**Per 1,000 CVC days**					
No documented assessment for CVC removal	616 (544 - 696)	411	253	140	371 (328 - 420)	668	248	222	0.68 (0.57 - 0.82)	< 0.001
	**Per 1,000 urinary catheter days**				**Per 1,000 urinary catheter days**					
No documented assessment for urinary catheter removal	715 (644 - 793)	494	353	168	360 (320 - 406)	750	270	255	0.56 (0.47 - 0.65)	< 0.001
	**Per 1,000 days of use**				**Per 1,000 days of use**					
No documented assessment to continue or discontinue current antimicrobials	294 (249 - 346)	477	140	163	154 (129 - 185)	759	117	262	0.67 (0.52 - 0.86)	0.002
No documented assessment to continue or discontinue current sedation	317 (264 - 382)	356	113	130	162 (132 - 200)	543	88	204	0.66 (0.50 - 0.87)	0.003

95%CI - 95% confidence interval; CVC - central venous catheter.

Table 11SB - Females: the incidence rates of omissions in daily care processes high–income countries

Total = 416 Observed event	Before, n = 155 Incidence rate (95%CI)	Observation days, n	Observed events, n	Patients, n	After, n = 261 Incidence rate (95%CI)	Observation days, n	Observed events, n	Patients, n	Adjusted for center effects incidence rate ratio	p value
	**Per 1,000 ventilator days**				**Per 1,000 ventilator days**					
No deep vein thrombosis prophylaxis	51.1 (29.0 - 89.9)	235	12	86	65.6 (45.6 - 94.4)	442	29	153	0.89 (0.45 - 1.76)	0.74
No peptic ulcer prophylaxis	12.8 (4.12 - 39.6)	235	3	86	6.79 (2.19 - 21.0)	442	3	153	0.48 (0.09 - 2.46)	0.38
No documented assessment of spontaneous breathing trial	460 (381 - 555)	235	108	86	258 (215- 310)	442	114	153	0.54 (0.41 - 0.70)	< 0.001
No documented family conference/ discussion	243 (187 - 314)	235	57	86	437 (379 - 503)	442	193	153	1.67 (1.22 - 2.27)	< 0.001
No daily oral care	68.1 (41.7 - 111.1)	235	16	86	47.5 (31.0 - 72.9)	442	21	153	0.73 (0.37 - 1.41)	0.36
No head of bed elevation at 30	17.0 (6.39 - 45.4)	235	4	86	4.52 (1.13 - 18.1)	442	2	153	0.19 (0.04 - 1.07)	0.051
	**Per 1,000 CVC days**				**Per 1,000 CVC days**					
No documented assessment for CVC removal	541 (463 - 632)	294	159	99	425 (373 - 485)	522	222	170	0.69 (0.56 - 0.85)	< 0.001
	**Per 1,000 urinary catheter days**				**Per 1,000 urinary catheter days**					
No documented assessment for urinary catheter removal	677 (596 - 770)	344	233	122	366 (321 - 417)	623	228	212	0.53 (0.44 - 0.64)	< 0.001
	**Per 1,000 days of use**				**Per 1,000 days of use**					
No documented assessment to continue or discontinue current antimicrobials	202 (160 - 255)	347	70	123	133 (108 - 164)	653	87	229	0.71 (0.52 - 0.98)	0.04
No documented assessment to continue or discontinue current sedation	319 (251 - 405)	210	67	86	185 (148 - 232)	411	76	156	0.52 (0.37 - 0.73)	< 0.001

95%CI - 95% confidence interval; CVC - central venous catheter.

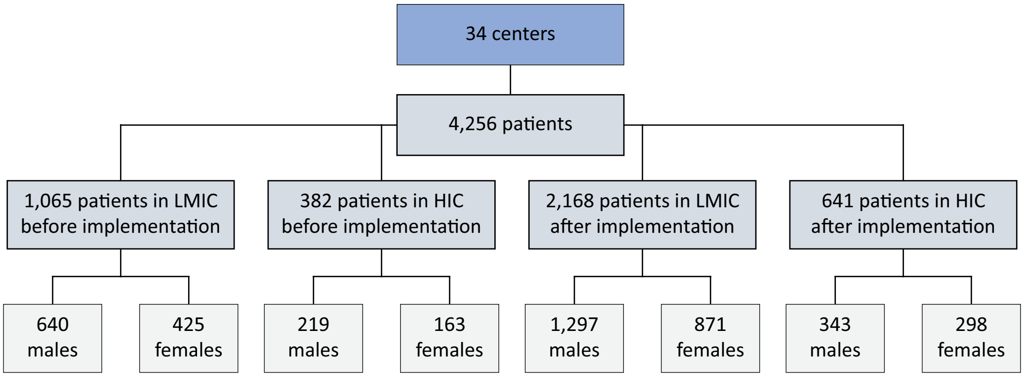

LMIC - low- and middle- income countries; HIC - high–income countries.

Figure 1S - Study flowchart.

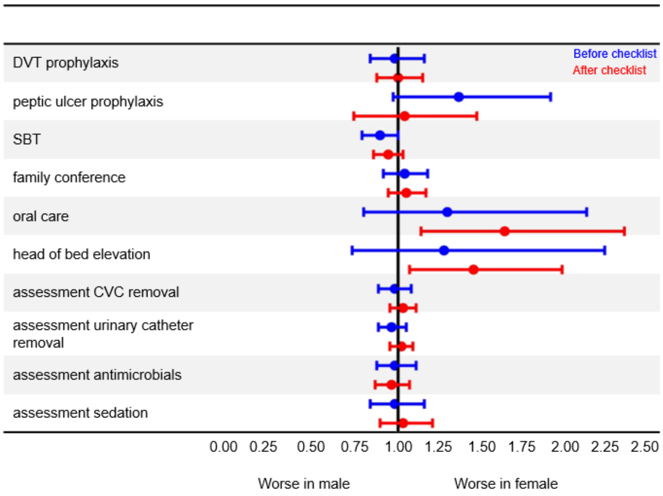

DVT - deep vein thrombosis; SBT - spontaneous breathing trials; CVC - central venous catheter.

Figure 2S - The incidence rate ratios of omissions in daily care processes between the sexes – low- and middle-income countries.
Before and after the CERTAIN implementation in low- and middle- income countries.

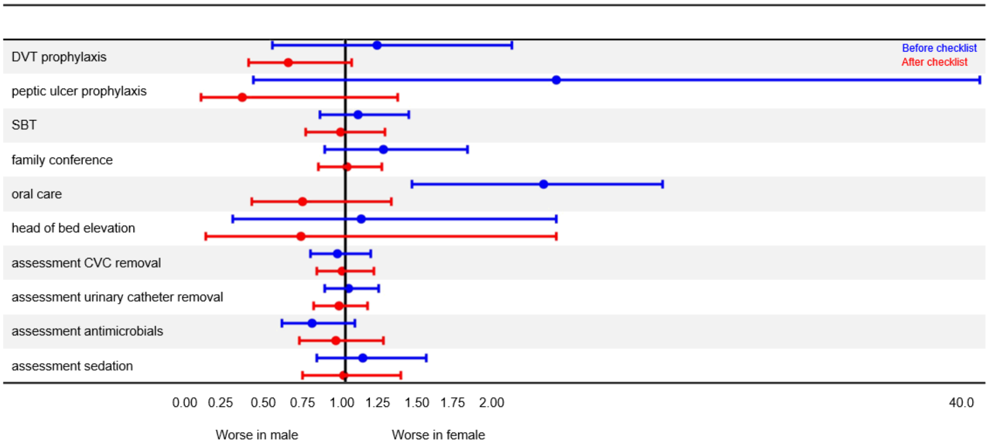

DVT - deep vein thrombosis; SBT - spontaneous breathing trials; CVC - central venous catheter.

Figure 3S - The incidence rate ratios of omissions in daily care processes between the sexes – high-income countries.
Before and after the CERTAIN implementation in high-income countries.

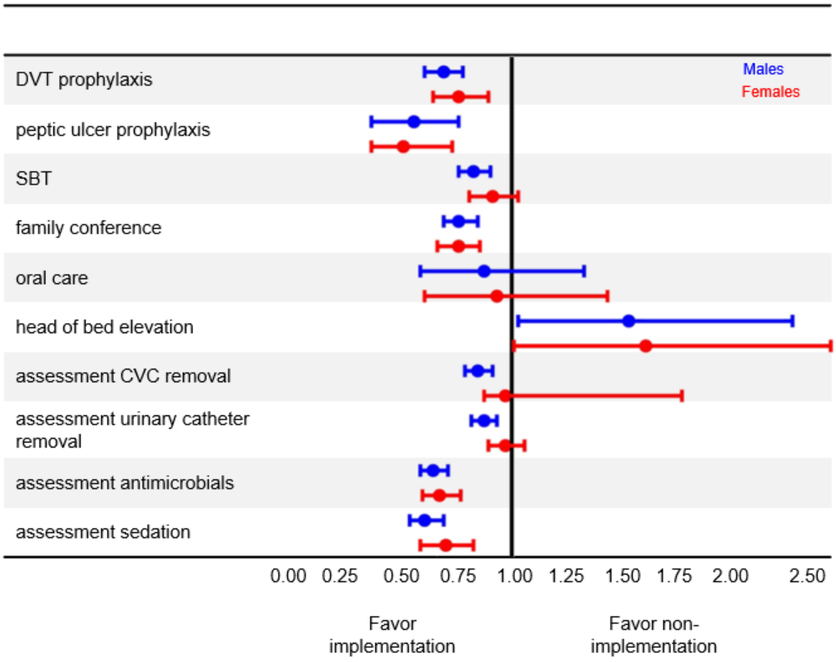

DVT - deep vein thrombosis; SBT - spontaneous breathing trials; CVC - central venous catheter.

Figure 4S - The incidence rate ratios of omissions in daily care processes before and after the implementation – low- and middle-income countries.
This shows the change in adherence to daily care processes before versus after the CERTAIN implementation in female and male patients in low- and middle-income countries.

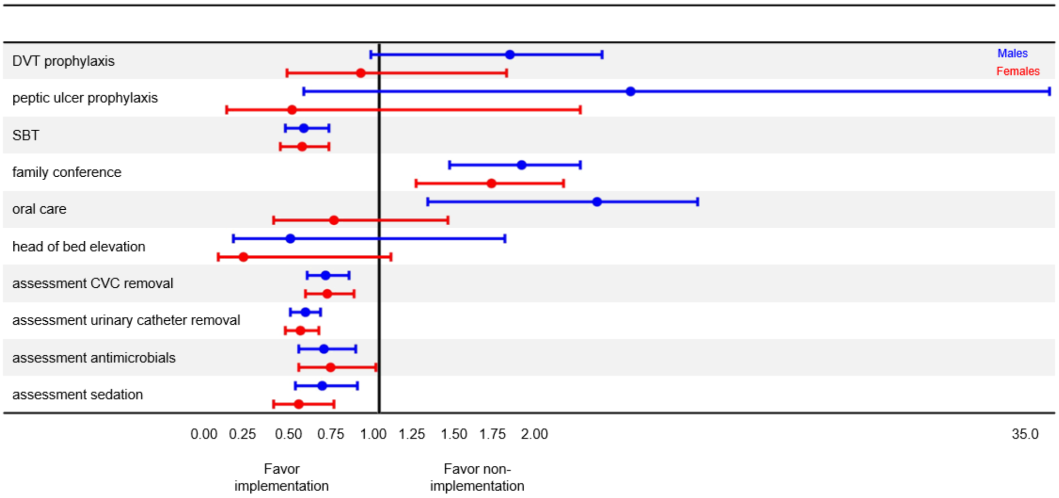

DVT - deep vein thrombosis; SBT - spontaneous breathing trials; CVC - central venous catheter.

Figure 5S - The incidence rates of omissions in daily care processes before and after the implementation – high-income countries.
This shows the change in adherence to daily care processes before versus after the CERTAIN implementation in female and male patients in high-income countries.
